# MHBSt^167^ induced autophagy promote cell proliferation and EMT by activating the immune response in L02 cells

**DOI:** 10.1186/s12985-022-01840-z

**Published:** 2022-06-27

**Authors:** Bin Cheng, Qiong Wang, Zhiqiang Wei, Yulin He, Ruiming Li, Guohua Liu, Shaobo Zeng, Zhongji Meng

**Affiliations:** 1 Institute of Biomedical Research, Hubei Clinical Research Center for Precise Diagnosis and Treatment of Liver Cancer, Taihe Hospital, Hubei University of Medicine, Shiyan, 442000 Hubei China; 2 Department of Infectious Diseases, Taihe Hospital, Hubei University of Medicine, Shiyan, 442000 Hubei China; 3 Department of Hepatobiliary Pancreatic Surgery, Taihe Hospital, Hubei University of Medicine, Shiyan, 442000 Hubei China; 4 Hubei Key Laboratory of Embryonic Stem Cell Research, Shiyan, 442000 Hubei China

**Keywords:** Hepatitis B virus, MHBSt^167^, Hepatocellular carcinoma, Immune response, Autophagy

## Abstract

**Background:**

Hepatitis B virus can induce hepatocellular carcinoma (HCC) by inducing a host immune response against infected hepatocytes. C-terminally truncated middle surface protein (MHBSt) has been reported to contribute to HCC through transcriptional activation in epidemiology studies, while the underlying mechanism of MHBSt-induced HCC is unknown.

**Methods:**

In this study, a premature stop at codon 167 in MHBS (MHBSt^167^) was investigated into eukaryotic expression plasmid pcDNA3.1(-). MHBSt^167^ expressed plasmid was transfected into the L02 cell line, cell proliferation was analyzed by CCK-8 and high-content screening assays, the cell cycle was analyzed by flow cytometry, and epithelial-to-mesenchymal transition and autophagy were analyzed by immunoblotting and immunofluorescence. NF-κB activation and the MHBSt^167^-induced immune response were analyzed by immunoblotting and immunofluorescence. IFN-α, IFN-β and IL-1α expression were analyzed by qPCR. Autophagy inhibitors were used to analyze the relationship between the immune response and autophagy.

**Results:**

The results showed that MHBSt^167^ promoted L02 cell proliferation, accelerated cell cycle progression from the S to G2 phase and promoted epithelial-to-mesenchymal transition through ER-stress, leading to autophagy and NF-κB activation and increased immune-related factor expression. The MHBSt^167^-induced acceleration of cell proliferation and the cell cycle was abolished by autophagy or NF-κB inhibitors.

**Conclusion:**

In summary, MHBSt^167^ could promote cell proliferation, accelerate cell cycle progression, induce EMT and activate autophagy through ER-stress to induce the host immune response, supporting a potential role of MHBSt^167^ in contributing to carcinogenesis.

**Supplementary Information:**

The online version contains supplementary material available at 10.1186/s12985-022-01840-z.

## Background

The development of hepatitis B virus (HBV)-related hepatocellular carcinoma (HCC) is polyfactorial, including cellular signaling pathway changes and cell cycle alteration, together with an inflammatory and cytokine responses that are driven by viral antigens [1], including HBV mutant variants. Among the HBV mutant variants, mutations in the S protein are common in patients and are related to HCC [2]. A previous study demonstrated that preS mutants were associated with an ascending risk (3.77-fold) of HCC and the forecast value of these mutants in the development of HCC had been defined [3, 4]. PreS2-defective viruses mutated in the promoter region or 5’-terminal regions are the main HBV variants and are more commonly associated with HCC. There have been few reports on the relationship between C-terminally truncated middle proteins and HCC, especially in vitro validation reports. Notably, preS/S sequences deleted at the 3’-end that produce functionally active C-terminally truncated middle surface protein (MHBSt) have been found in many HBV DNA positive HCC patients, and protein kinase C (PKC)-dependent activation of the c-Raf-1/MEK/Erk2 signaling pathway was triggered by MHBSt retained in the endoplasmic reticulum (ER) in MHBSt transgenic mice and hepatoma cells, which, leading to regulation of AP-1 and enhanced proliferative activity of hepatocytes [5]. MHBSt act as a transcriptional activator to activating the hTERT promoter in hTERT highly expressed preS2-positive human HCC samples, leading to upregulated telomerase activity and promoting HCC development [6]. MHBSt^167^ was one of the first reported mutations of S-truncated proteins, and MHBst^167^/HBx could induce nuclear factor-κB (NF-κB) activation via the PKC/ERK pathway in renal tubular cells [7, 8]. The transcriptional activity of the c-Myc promoter could be upregulated by MHBst^167^, as well as transcription and translation of c‑Myc, which is a proto‑oncogene, in HepG2 cells [9]. The relationship between MHBSt^167^ and HCC is unclear, and the mechanism remains to be determined.

Autophagy is a fundamental process of cells which eliminates damaged intracellular organelles and misfolded proteins to maintain cellular homeostasis [10]. The role of autophagy in the liver is complex. The liver requires autophagy to remove excessive aggregated proteins, accumulated lipids, and impaired mitochondria to prevent excessive production of reactive oxygen species (ROS), which leads to oxidative stress in the ER [11]. Unresolved oxidative stress, persistent inflammation, and viral infections are the most commonly identified risk factors for HCC development. ER-induced autophagy plays a protective role against both initial and persistent liver injury and a vital role in the development and growth of hepatic tumor cells in an inflammatory environment [12, 13]. MHBSt can be retained in the ER and trigger ER stress [5]. The antioxidants N-acetyl-L-cysteine (NAC) and pyrimidine dithiocarbamate (PDTC) can block host gene induction by MHBSt, indicating that MHBSt can induce oxidative stress, which is a risk factor for HCC development [8]. Based on the above literature, we hypothesized that MHBSt could induce autophagy to promote HCC development.

Most cases (80–90%) of liver cancer arise in the setting of a chronically inflamed liver (due to hepatitis B, hepatitis C, or alcoholic and nonalcoholic liver diseases) or liver fibrosis/cirrhosis, HCC can be considered a prototype of inflammation-derived cancer arising from chronic liver injury [14]. In chronic liver disease, innate immune response plays a critical role in persistent inflammation, fibrosis/cirrhosis, and the persistently accelerated turnover of liver cells provides the basis for the occurrence of liver cancer. NF-κB is well accepted as a central mediator that regulates immune and inflammatory responses [15]. MHBSt is produced by nonintegrated viral variants to cope with the selective pressure of the host immune response [16]. MHBSt can cause DNA binding activity of NF-κB [8]. These results indicate that the immune response may be associated with MHBSt. Autophagy participates in most intracellular stress response pathways, including immune response and inflammation control pathways [17]. These interactions act both at changing the autophagy level and regulating direct interactions between autophagy proteins and immune signaling molecules [18]. Autophagy pathways/proteins, immunity and inflammation can be interregulated through positive and negative feedback [19], suggesting that MHBSt-induced autophagy and the immune response may interact reciprocally to regulate HCC development.

In the present study, *MHBSt*^167^ was expressed in the L02 cell line, and the oncogenicity of MHBSt^167^ was analyzed. The expression of autophagy-related proteins and the activation of NF-κB were examined. Autophagy inhibitors were used to analyze whether autophagy and the immune response induced by MHBSt^167^ interact with each other to regulate HCC development.

## Materials and methods

### Chemicals and reagents

Dulbecco’s modified Eagle’s medium (DMEM), fetal bovine serum (FBS), Lipofectamine^™^ 3000 transfection reagent, TRIzol reagent, LysoTracker Red and the high capacity cDNA reverse transcription kit were purchased from Thermo Fisher Scientific (Waltham, MA, USA). Rapa and 3-MA were obtained from Sigma-Aldrich Co. (St Louis, MO, USA). CQ and BAY-11-7082 were obtained from Selleck (Houston, Texas, USA). Mouse anti-human monoclonal antibodies against PreS2/S, β-actin, and histone 3 were purchased from Abcam (C ambridge, UK). Anti-human monoclonal antibodies against LC3, Beclin-1, SQSTM/P62, NF-κB, p-NF-κB/p65, IκB, p-IκB, Vimentin, E-cadherin and Protein Disulfide Isomerase (PDI) were purchased from Cell Signaling Technology (Boston, MA, USA). The Immobilon Western Chemiluminescent HRP substrate was purchased from EMD Millipore (Billerica, MA, USA). The cell counting kit-8 was purchased from Beyotime (Shanghai, China). Alexa Fluor 488-conjugated goat anti-mouse IgG (H + L), Alexa Fluor 594-conjugated goat anti-rabbit IgG (H + L), DyLight 405-labeled goat anti-mouse IgG (H + L) and Alexa Fluor 488-conjugated goat anti-rabbit IgG (H + L) were purchased from ZSGB-BIO (Beijing, China). Propidium Iodide (PI)/RNase staining buffer was purchased from BD Biosciences (Franklin, NJ, USA).

### Plasmid construction

To construct the pcDNA3.1-MHBSt^167^ and pcDNA3.1-MHBS plasmids, the genes were amplified from the Phy106 + wta plasmid (HBV adr genome). The primers were as follows: *MHBSt*^*167*^ and *MHBS* forward primer, 5’GAATTCATGCAGTGGAACTCCACAAC3’; *MHBSt*^167^ reverse primer, 5’CTGCAGCTATCCTGGAAGTAGAGGACAAAC3’, and *MHBS* reverse primer, 5’CTGCAGTTAAATGTATACCCAAAGAAAATTGG3’. These two ligated vectors were confirmed by DNA sequence analysis. The primers for IFNα, IFNβ and IL-1α were purchased form QIAGEN (Dusseldorf, Germany). The GFP-LC3 and mRFP-GFP-LC3 plasmids were purchased from Addgene.

### Cell culture

The human hepatocyte cell line L02 was obtained from the Chinese Academy of Sciences Cell Bank (Shanghai, China) and cultured in DMEM with 10% FBS and 1% penicillin–streptomycin (10,000 U/mL penicillin and 10 mg/mL streptomycin) at 37 °C in an incubator containing 5% CO_**2**_ and moderate amount of water vapor. Change the medium every two days, and cell passage or subsequent experiments was performed when they grew to 80% density.

### cDNA synthesis and PCR

After being harvest, cells were washed with ice-cold PBS (Phosphate Buffer Saline) for twice in plates, and TRIzol reagent was added into plates to extract total RNA according to the manufacturer’s instructions. RNA concentration and purity were measured using a UV spectrophotometer (Eppendorf, Hamburg, Germany). Synthesize cDNA was performed by using the high capacity cDNA reverse transcription kit according to the manufacturer’s instructions. Then, PCR was followed. The primers used for PCR were the same as those used to construct the pcDNA3.1-MHBSt^167^ and pcDNA3.1-MHBS plasmids.

### Immunoblotting

After being harvest, cells were washed with ice-cold PBS for twice and total protein was extract by RIPA lysis buffer according standard procedure. The protein concentration was determined by bicinchoninic acid analysis according to the manufacturer’s instructions. Homogenized protein extract and were separated by sodium dodecyl sulfate polyacrylamide gel electrophoresis (SDS-PAGE) and then transferred onto nitrocellulose membranes. Blocking the membranes with 5% skim milk for 2 h and then incubated with primary antibodies diluted at a ratio of 1:1,000 with 3% skim milk overnight at 4 °C. Washing the membranes with Tris-buffered saline with Tween-20 for 3 times to remove the unbound primary reactance, and then the membranes were incubated with HRP-conjugated secondary antibodies at room temperature for 2 h. An enhanced chemiluminescent kit was used to visualize the immunoreactive proteins according to the manufacturer’s protocol.

### Cell counting kit-8 (CCK-8) assay

CCK-8 assays were performed according to the manufacturer’s instructions. Briefly, L02 cells were seeded in 96-well plates at 2 × 10^4^ cells/well and transfected with the MHBS and MHBSt^167^ expression plasmids. Before harvest, CCK-8 solution was added into each well and incubated for 2 h at 37 °C. Finally, the absorbance of the lysates was measured at 570 nm using a microplate reader.

### Cell cycle analysis

PI/RNase staining buffer was used to analyze the cell cycle as previously reported. After L02 cells were transfected with pcDNA3.1-MHBSt^167^ and pcDNA3.1-MHBS plasmids for 48 h, the cells were washed with cold PBS, harvested, fixed with 70% ethanol at − 20 °C for 1 h, and centrifuged at 1000 g for 10 min at 4 °C. The cells were then washed with cold PBS and resuspended in PI/RNase staining buffer for 15 min in the dark, followed by FCM analysis.

### Colony formation assay

Colony formation assay is an important method to detect cell proliferation ability. In 6-well plate, when a single cell proliferates for more than 50 cells (about 7–14 d), they become a clone with a size between 0.3 and 1.0 mm. The proliferation ability of L02 cells was monitored by plate cloning assay. Cell suspensions (2000 cells/ well) were prepared and seeded in 6-well plate, which were then transfected with Vector, MHBS and MHBSt^167^ for 7–14 d. The visible cell colonies were fixed with 4% paraformaldehyde and stained with crystal violet solution for 15 min. Typical colony images were recorded with camera and Microscopic imaging system, and the number of cell colonies was counted.

### Immunofluorescence and confocal microscopy

Cells were transfected with *MHBSt*^*167*^ and *MHBS* expression plasmids using Lipofectamine 3000. Forty-eight hours after transfection, LysoTracker Red was added to the medium and incubated for 2 h at 37 °C. The cells were washed with PBS, and then fixed in 4% paraformaldehyde, and permeabilized with 1% Triton X-100. The cells were washed with PBS three times. For immunofluorescence analysis, the cells were blocked with FBS for 30 min and incubated with the appropriate primary antibodies and fluorescently labeled secondary antibodies, followed by fluorescently-labeled secondary antibodies. Then, DAPI (Sigma) was included in the final wash at a final concentration of 0.1 μg/mL to stain the nuclei. The images were visualized with confocal microscopy. The resulting images were deconvolved with Delta vision software.

#### Statistical analysis

The data are presented as the mean ± standard error mean. Experiments were repeated at least two times. A two-way chi-square test was used for cell cycle data analysis. Student’s t-test was used for the rest of data analysis. All data analysis was performed by using GraphPad Prism7 software. A value of P < 0.05 and P < 0.01 were considered to be statistically significant.

## Results

### MHBSt^167^ promoted cell proliferation and accelerated the cell cycle from the S phase to the G2/M phase and induced epithelial-to-mesenchymal transition (EMT) in L02 cells

**Fig. 1:**
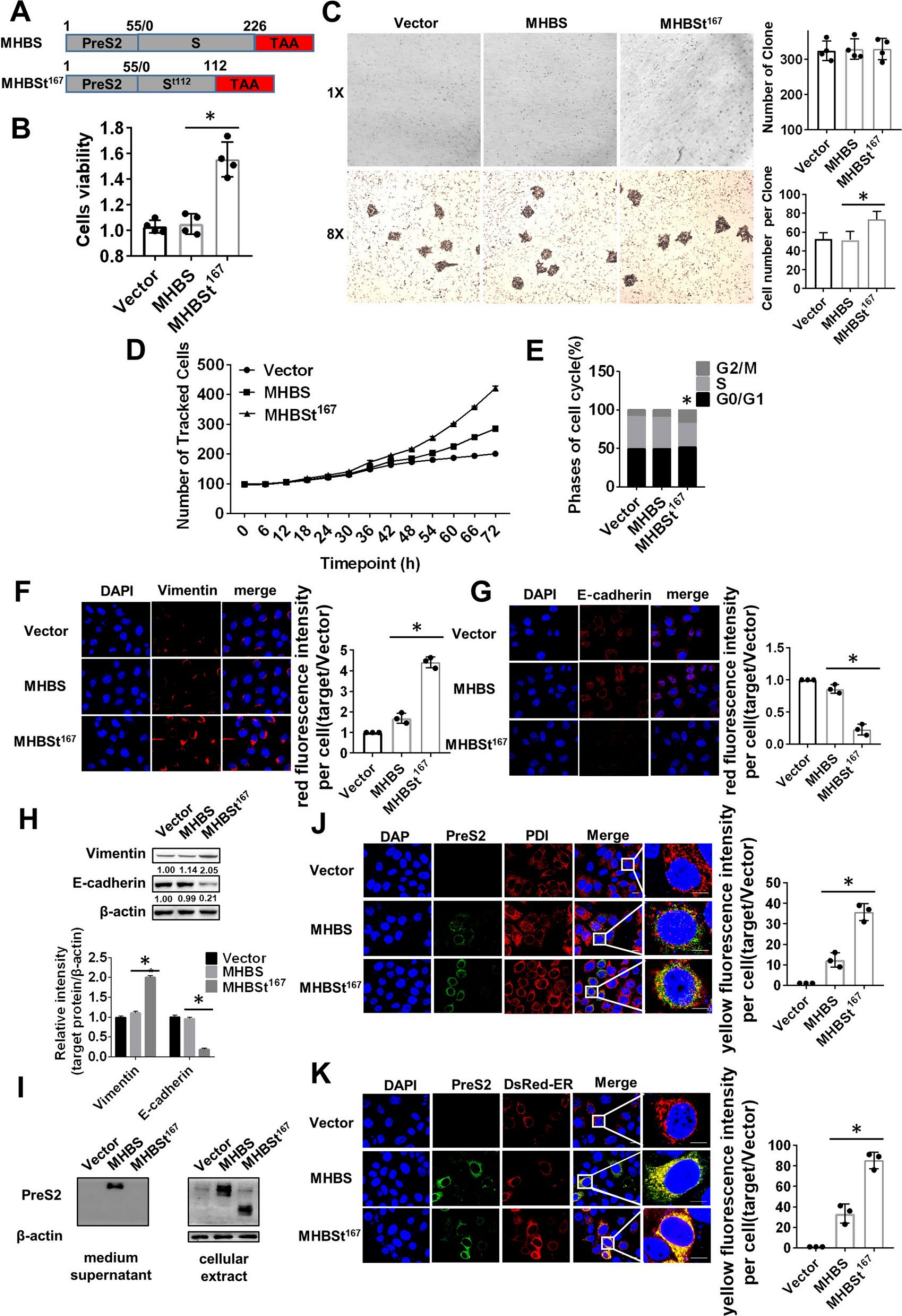
Effects of MHBSt^167^ expression on cell proliferation, cell cycle and EMT in L02 cells. **A** Structural diagrams of plasmids. **B** Cell viability was measured by CCK-8 assays after being transfected with plasmids for 48 h. The results are expressed as OD values, *P < 0.05. **C** Cell proliferation ability was measured by clone formation assay transfected with plasmids for 7–10 days. The clone number per well and cell number per clone were analyzed. **D** Cell proliferation was measured by high-content screening (HCS) assay. The results are expressed as cell count values every 0.5 h. **E** Cell cycle was analysed by flow cytometry. Cells were stained with PI after transfection for 48 h. **F**, **G** Confocal microscopic analysis of Vimentin and E-cadherin by immunofluorescence staining. Nuclei were stained with DAPI. The red fluorescence intensity per cell transfected with Vector was defined as 1. **H** Western blotting analysis of Vimentin and E-cadherin protein expression. The individual gray value in Western blots was measured and normalized against β-actin, then the relative intensity of target protein to β-actin was calculated by setting the control vector transfection as 1.00, *P < 0.05. **I** Western blotting analysis of MHBSt^167^ in culture supernatants and cellular extracts. **J** Confocal microscopic analysis of the colocalization of MHBSt^167^ and the ER. The ER was labeled with PDI for immunofluorescence staining with an anti-PDI antibody. Nuclei were stained with DAPI. The fluorescence intensity per cell in cells transfected with Vector was de-fined as 1. **K** Confocal microscopic analysis of the colocalization of MHBSt^167^ and the ER. Exogenous DsRed-ER expression plasmids were transfected into cells. Nuclei were stained with DAPI. The fluorescence intensity per cell in cells transfected with Vector was defined as 1. Scale bar: 50 μm. Representative images from three independent experiments are shown

A premature stop at codon 167 in MHBs was introduced to create the protein MHBSt^167^ (Fig. 1A). DNA sequencing results indicated that the recombinant plasmid contained the HBV DNA fragment encoding the truncated middle surface protein that was in‑frame without any base mutations. MHBSt^167^ mRNA expression in L02 cells was successfully detected by reverse transcription polymerase chain reaction (RT‑PCR) (Additional file 1: Fig. S1A). Different amounts of MHBS and MHBSt^167^ plasmids were transfected into cells to ensure consistent levels of protein expression, and 1 (pcDNA3.1-MHBS):3 (pcDNA3.1-MHBSt^167^) was the best ratio (Additional file 1: Fig. S1B). CCK-8 assays, clone formation assay and high-content screening (HCS) assays were used to examine MHBSt^167^-induced cell proliferation. The results revealed that L02 cells transfected with MHBSt^167^ showed 50.43 ± 7.85% higher viability than cells transfected with MHBS, and Hep3B, LM3, Huh7, and HepG2 cells have the similar phenotypes as in L02 cells, the cell number per clone in L02 cells transfected with MHBSt^167^ is 30% larger than in cells transfected with MHBS (P < 0.05, Fig. 1B–D, Additional file 1: Fig. S1C–F). The flow cytometry (FCM) results showed that the proportion of cells in the G2/M phase was increased 75.93%, while the proportion of cells in the S phase was decreased 29.55% in cells transfected with MHBSt^167^ compared with those transfected with MHBS (P < 0.05, Fig. 1E, Additional file 1: Fig. S1G). In human cancer, the activation of the EMT process is related to advanced disease [20], which was demonstrated by the transition of epithelial biomarkers (E-cadherin) to mesenchymal biomarkers (Vimentin). Therefore, the effect of MHBSt^167^ on the EMT process in L02 cells was investigated by immunoblotting and immunofluorescence. The immunofluorescence showed increased Vimentin expression levels and decreased E-cadherin expression levels in cells transfected with MHBSt^167^and the immunoblotting results showed that cells transfected with MHBSt^167^ showed 80.81 ± 2.60% upregulated Vimentin expression levels and 80.20 ± 1.57% downregulated E-cadherin expression levels than cells transfected with MHBS, and Hep3B, LM3, Huh7, and HepG2 cells have the similar phenotypes as in L02 cells (P < 0.05, Fig. 1F–H, Additional file 1: Fig. S1H). To explore the pathway that MHBSt changing cell cycle and promoting cell proliferation, MHBSt^167^ expression in medium supernatant and cellular extracts were detected and co-location of MHBSt^167^ and ER marker protein was detected. The results showed that MHBSt^167^ was only detected in cellular extracts, and MHBS was detected both in cell culture supernatants and cellular extracts (Fig. 1I and Additional file 1: Fig. S1I). Protein Disulfide Isomerase (PDI) catalyzes internal disulfide bond exchange and promote formation of correct disulfide bonds and is a marker of ER. PDsRed-ER is a mammalian expression vector used to label the endoplasmic reticulum of living cells. Immunofluorescence showed threefold higher co-location of MHBSt^167^ and Protein Disulfide Isomerase (PDI) and 3.5-fold higher co-location of MHBSt^167^ and DsRed-ER in cells transfected with MHBSt^167^ than in cells transfected with MHBS (Fig. 1J, K).

### Complete autophagic flux was induced by MHBSt^167^ in L02 cells

**Fig. 2:**
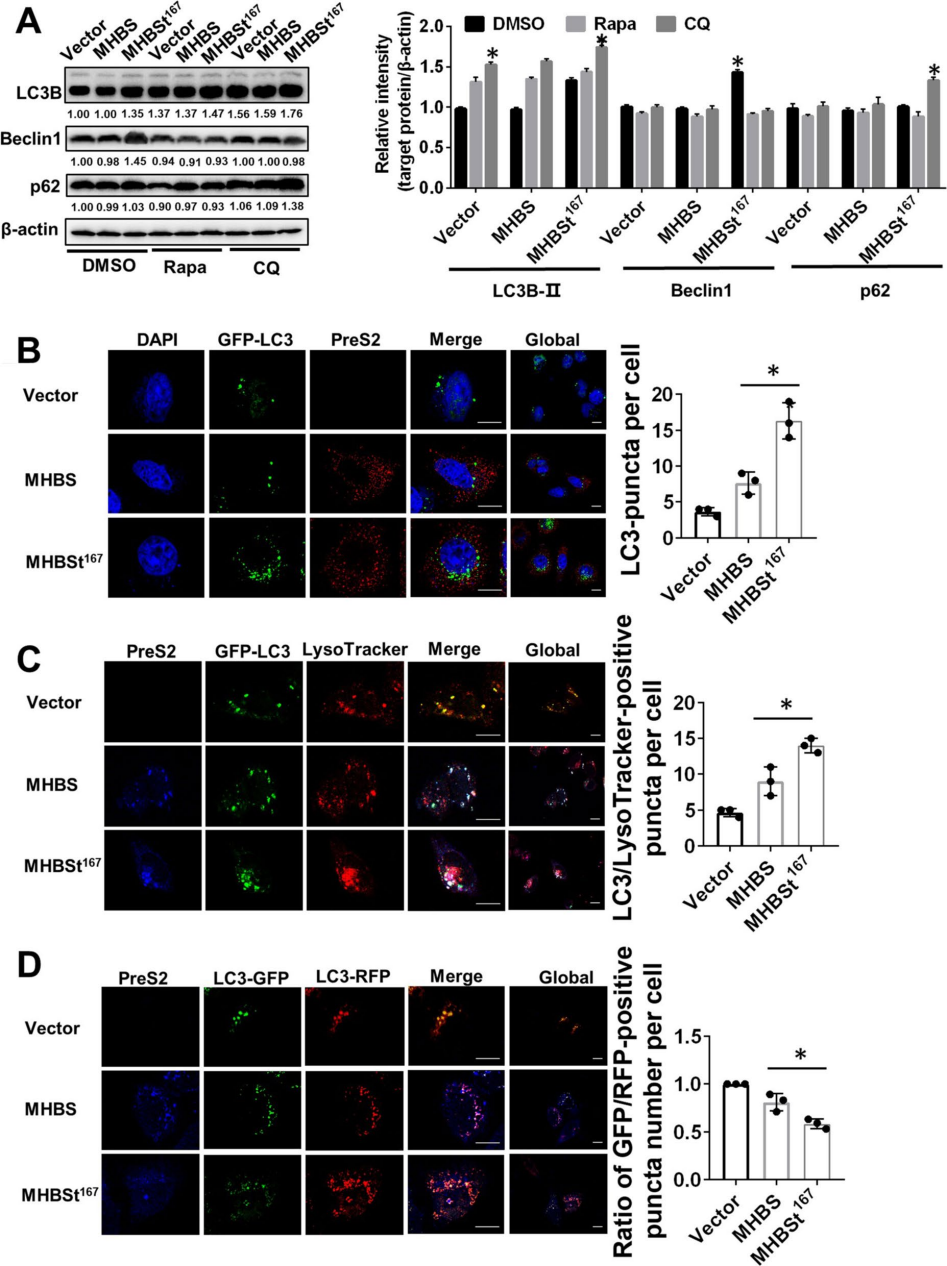
Effects of MHBSt^167^ expression on autophagy in L02 cells. **A** Western blotting analysis of autophagy-related proteins (LC3B, Beclin1, and p62) and statistical analysis. Cells were treated with DMSO, 100 nM rapamycin (Rapa), or 4 mM chloroquine (CQ) for 2 h and then transfected with MHBS and MHBSt^167^ expression plasmids for 48 h. The individual gray value in Western blots was measured and normalized against β-actin, then the relative intensity of target protein to β-actin was calculated by setting the control vector transfection as 1.00, *P < 0.05. **B** Confocal microscopic analysis of LC3B puncta induced by MHBSt^167^ via immunofluorescence staining. An exogenous GFP-LC3 expression plasmid was transfected into L02 cells. Nuclei were stained with DAPI. The LC3-puncta per cell was analyzed. **C** Confocal microscopic analysis of autophagic lysosome formation induced by MHBSt^167^. An exogenous GFP-LC3 expression plasmid was transfected into L02 cells. Before immunofluorescence staining, the cells were incubated with 50 nM LysoTracker Red for 2 h. The LC3/LysoTracker-positive puncta per cell was analyzed. **D** Confocal microscopic analysis of autophagy flux induced by MHBSt^167^. An exogenous RFP-GFP-LC3 expression plasmid was transfected into L02 cells. The ratio of GFP/RFP–positive puncta number per cell was analyzed. The ratio in cells transfected with Vector was defined as 1. Scale bar: 50 μm. Representative images from three independent experiments are shown

To explore whether autophagy was induced by MHBSt^167^, autophagy-related proteins were measured by immunoblotting. The results showed that the expression of MHBSt^167^ led to 36.01 ± 2.51% increased expression of LC3B-II and 45.00 ± 2.36% increased expression of beclin1 compared to the vector expressing cells. However, the expression of p62 protein, which is an autophagy receptor, has no change in MHBSt^167^ expressing cells. The autophagy activator rapamycin (Rapa) and autophagy inhibitor chloroquine (CQ) were used to analyze whether complete autophagic flux was induced by MHBSt^167^. After Rapa treatment, the expression of LC3B-II was slightly increased and p62 protein level was slightly decreased. However, rapamycin treatment leaded to 35.86 ± 2.33% decreased expression of Beclin1 in MHBSt^167^expressing cells. After CQ treatment, however, LC3B-II expression was significantly increased, as was p62 protein expression (P < 0.05, Fig. 2A). These results showed that MHBSt^167^ could induce complete autophagic flux. To verify this conclusion, exogenous GFP-LC3 and GFP-RFP-LC3 expression plasmids and LysoTracker Red were used to analyze the autophagy level by confocal microscopy. There was distinct accumulation of GFP puncta at 48 h after being co-transfected with exogenous GFP-LC3 and MHBSt^167^, and the number of GFP puncta was greater than those in cells co-transfected with exogenous GFP-LC3 and MHBS (Fig. 2B). These results indicated that MHBSt^167^ could induce enhanced autophagosome formation. LysoTracker Red is a specific lysosomal probe that can label lysosomes and fluoresce red. Cells were incubated with LysoTracker Red for 30 min before being harvested, and then the cells were immunofluorescently stained with PreS2. The results showed that more red fluorescence and green fluorescence co-localized in MHBSt^167^-expressing cells (Fig. 2C), indicating that autophagosomes and lysosomes were fused to produce autolysosome. The GFP-RFP-LC3 expression plasmid was used to mark and track LC3; in this system, green fluorescence will decrease or disappear in functional autolysosome. The GFP-RFP-LC3 expression plasmid was co-transfected with MHBSt^167^, and cells were immunofluorescently stained with PreS2. Statistical analysis showed that the ratio of fluorescence intensity (GFP/RFP) was lower in MHBSt^167^ expressed cells than in MHBS expressed cells, which indicate that more functional autolysosomes were formed in MHBSt^167^ expressing cells than in MHBS expressing cells (Fig. 2D). These results showed that MHBSt^167^ could induce much higher autophagy levels than MHBS.

### MHBSt^167^-induced autophagy promoted cell proliferation and accelerated cell cycle progression from S phase to G2/M phase in L02 cells

**Fig. 3:**
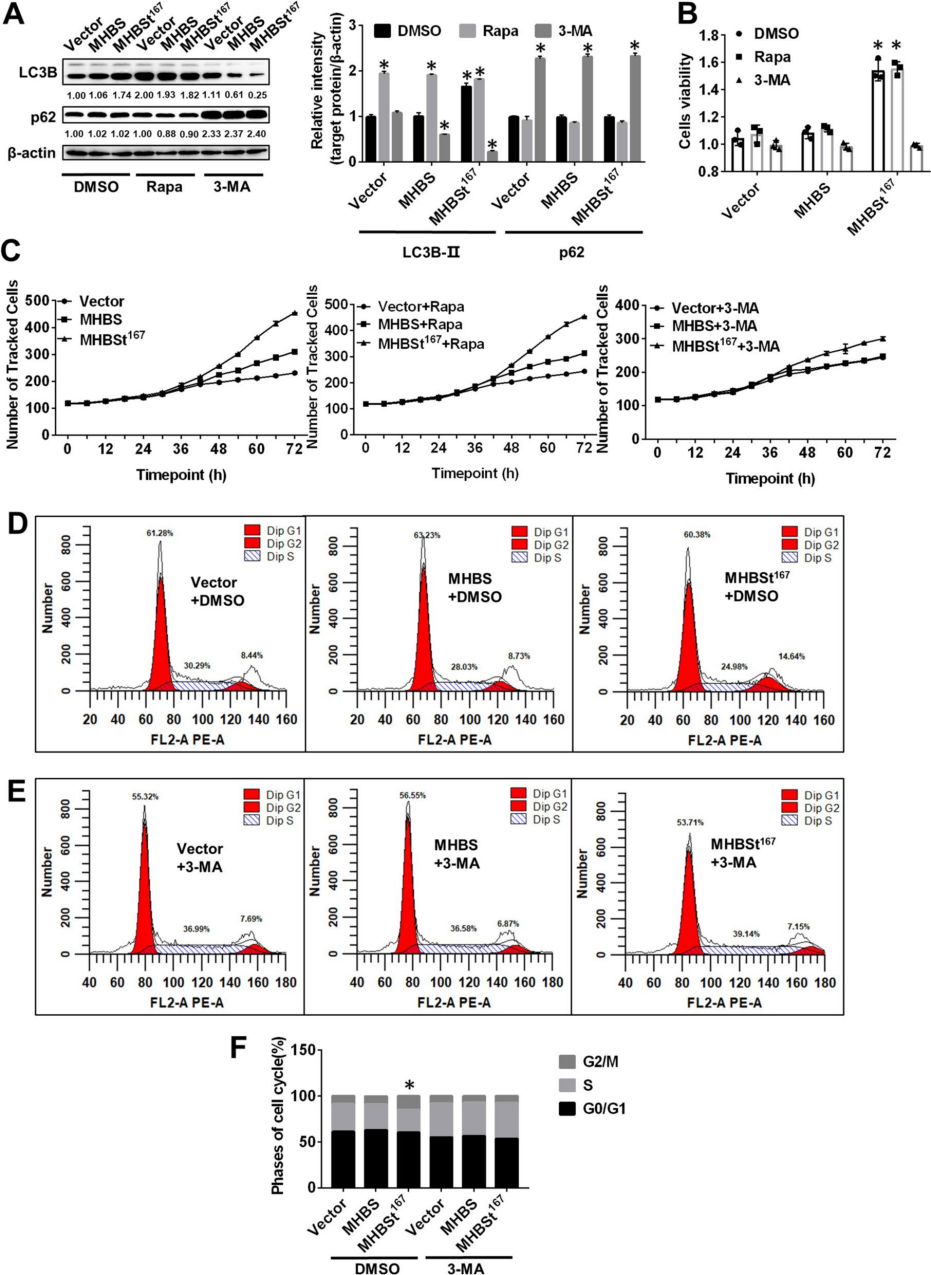
Effects of autophagy induced by MHBSt^167^ on cell proliferation and the cell cycle in L02 cells. **A** Western blotting analysis of autophagy-related proteins (LC3B and p62) and statistical analysis. Cells were treated with DMSO, 100 nM rapamycin (Rapa), and 2 mM 3-MA for 2 h and then transfected with MHBS and MHBSt^167^ expression plasmids for 48 h. The individual gray value in Western blots was measured and normalized against β-actin, then the relative intensity of target protein to β-actin was calculated by setting the control vector transfection as 1.00, *P < 0.05. **B** Cell viability was measured by CCK-8 assays in L02 cells. Before transfection with the MHBSt^167^ expression plasmid, cells were treated with DMSO, 100 nM Rapa, and 2 mM 3-MA for 2 h. The results are expressed as the OD values from three experiments performed in duplicate. *P < 0.05. **C** Cell proliferation was measured by high-content screening (HCS) assays. The results are expressed as cell count values obtained every 0.5 h for 72 h. **D**–**F** Cell cycle analysis was performed by flow cytometry. Cells were harvested after being transfected with plasmids for 48 h and stained with PI. Representative images from three independent experiments are shown

Autophagy has been increasingly shown to play a role in HCC. To explore whether MHBSt^167^-induced autophagy contributes to HCC, the autophagy activator Rapa and the autophagy inhibitor 3-methyl adenine (3-MA) were used. The results showed that Rapa treatment leaded to 16.33 ± 4.32% increased expressed level of LC3B-II and 12.58 ± 3.71% decreased expressed level of p62, while 3-MA treatment leaded to 86.69 ± 0.46% decreased expressed level of LC3B-II and 137.04 ± 4.87% increased expressed level of p62 in MHBSt^167^-expressing cells (P < 0.05, Fig. 3A). The CCK-8 assay showed that the increase in cell proliferation induced by MHBSt^167^ was scarcely changed by Rapa treatment, while cell proliferation was abolished by 3-MA treatment (P < 0.05, Fig. 3B). An HCS assay confirmed that the autophagy inhibitor abolished the increase in cell proliferation (Fig. 3C). The cell cycle was analyzed by FCM, and the results showed that the increase in the proportion of G2/M-phase cells after transfection with MHBSt^167^ was abolished by 3-MA treatment (Fig. 3D, E, F), suggesting that MHBSt^167^-induced autophagy accelerated cell cycle progression from S phase to G2/M phase.

### NF-κB, a central mediator that regulates the immune response, was activated by MHBSt^167^

**Fig. 4:**
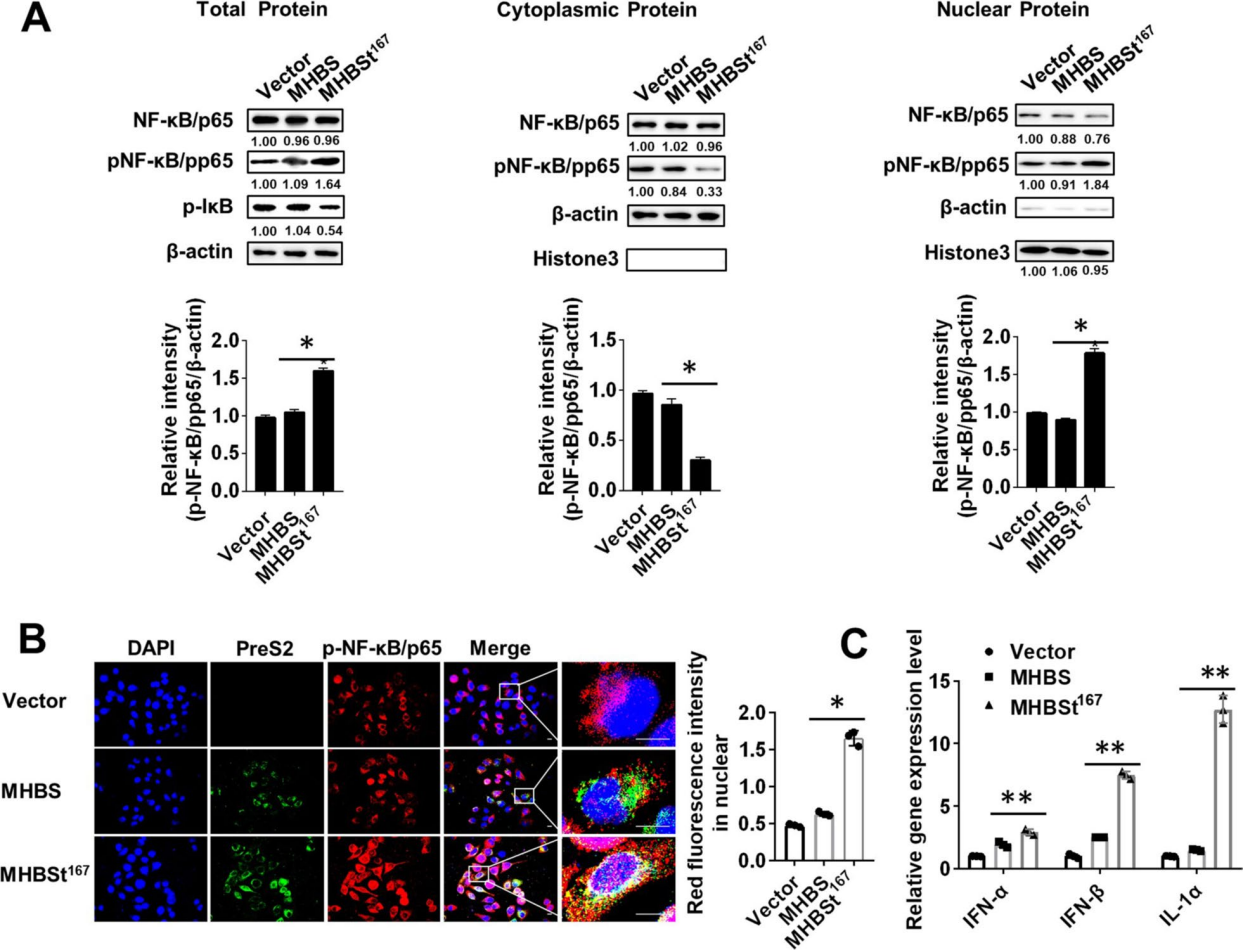
Effects of MHBSt^167^ expression on NF-κB activation in L02 cells. **A** Western blotting analysis of NF-κB activation and statistical analysis. The individual gray value in Western blots was measured and normalized against β-actin, then the relative intensity of target protein to β-actin was calculated by setting the control vector transfection as 1.00, *P < 0.05. **B** Confocal microscopic analysis of NF-κB activation. The intracellular localization of p-NF-κB/p65 was determined by immunofluorescence staining using an anti-p-NF-κB/p65 antibody. Nuclei were stained with DAPI. The red fluorescence intensity in unclear was analyzed by Image J. *P < 0.05. Scale bar: 50 μm. **C** RT-PCR analysis of IFN-α, IFN-β and IL-1α in cells. **P < 0.01. Representative images from three independent experiments are shown

NF-κB is now well accepted as a central mediator that regulates immune and inflammatory responses. The translocation of phosphorylated NF-κB/p65 (p-NF-κB/p65) from the cytoplasm to the nucleus represents the activation of NF-κB. To investigate whether MHBSt^167^ induces the host immune response, NF-κB activation was measured in MHBSt^167^ expressing cells. The immunoblotting results showed that p-NF-κB/p65 was 51.43 ± 3.59% increase in the total protein and 81.10 ± 9.00% increase in nuclear protein in MHBSt^167^ expressing cells, and it was 55.33 ± 4.29% decreased in the cytoplasmic fraction (P < 0.05, Fig. 4A). In addition, the expression level of p-IκB, a NF-κB inhibited element, was 44.52 ± 4.45% decreased. The immunofluorescence results showed that p-NF-κB/p65 was mainly located in the cytoplasm in cells expressing MHBS, while it exhibited less expression in the cytoplasm but much more expression in the nucleus in cells expressing MHBSt^167^; thus, p-NF-κB/p65 was transferred from the cytoplasm to the nucleus in MHBSt^167^-expressing cells, which indicated that NF-κB was activated (Fig. 4B). The RT-PCR results showed that the expression of IFN-α, IFN-β and IL-1α in cells expressing MHBSt^167^ was upregulated compared to that of MHBS-expressing cells, which means the innate immunity was activated by MHBSt^167^ (P < 0.01, Fig. 4C).

### MHBSt^167^-induced NF-κB activation promoted cell proliferation and accelerated cell cycle progression from S phase to G2/M phase in L02 cells

**Fig. 5:**
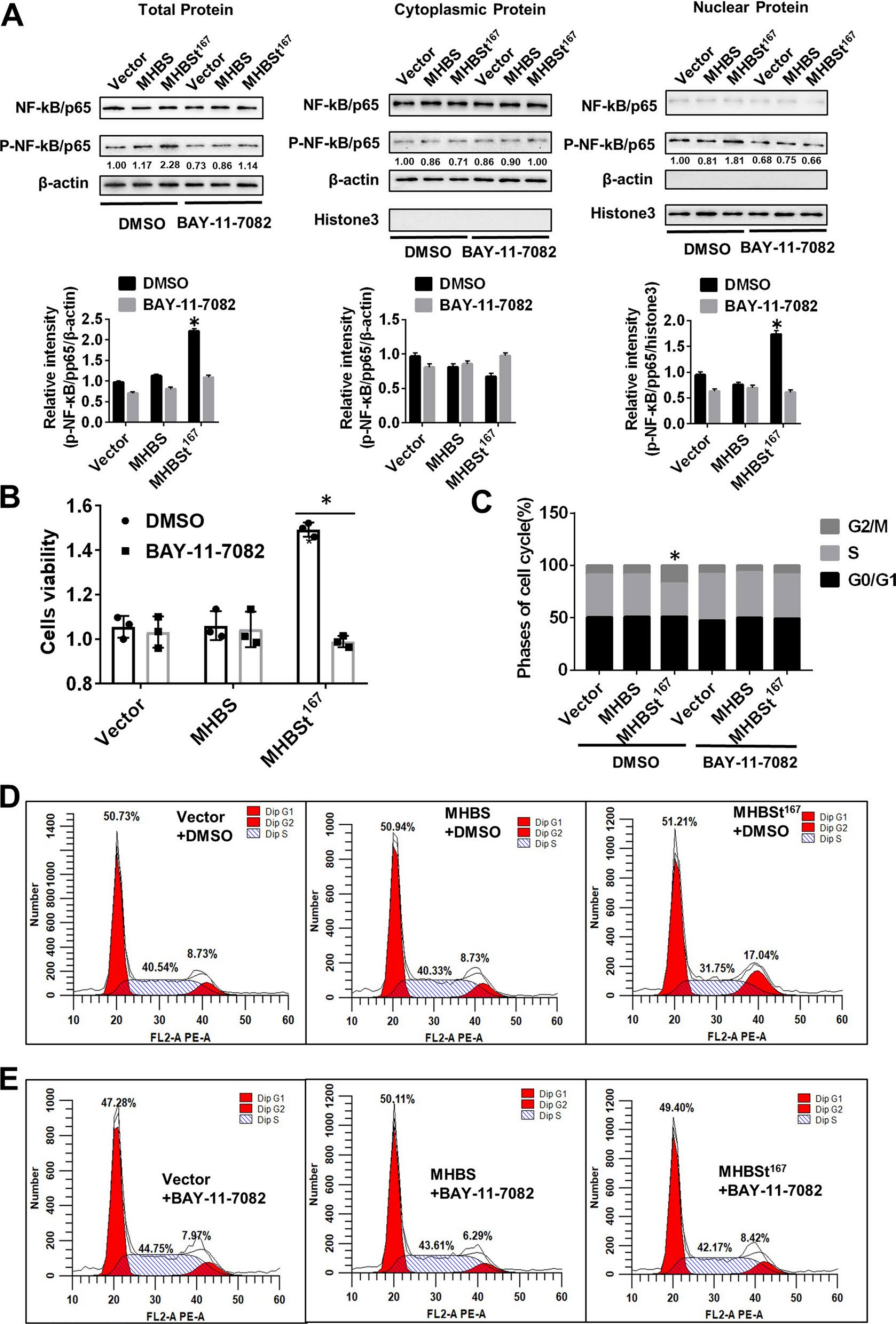
Effects of MHBSt^167^-induced NF-κB activation on cell proliferation and the cell cycle in L02 cells. **A** Western blotting analysis of NF-κB activation and statistical analysis. Before transfection with MHBS and MHBSt^167^ expression plasmids, cells were treated with DMSO and 0.3 µM BAY-11-7082 for 2 h. The individual gray value in Western blots was measured and normalized against β-actin, then the relative intensity of target protein to β-actin was calculated by setting the control vector transfection as 1.00, *P < 0.05. **B** Cell viability was measured by CCK-8 assay in L02 cells. Before transfection with MHBS and MHBSt^167^ expression plasmids, cells were treated with DMSO and 0.3 µM BAY-11-7082 for 2 h. The results are expressed as the OD values. *P < 0.05. **C**–**E** Cell cycle analysis was performed by flow cytometry. Cells were harvested after being transfected with plasmids for 48 h and stained with PI. Representative images from three independent experiments are shown

To explore the effect of MHBSt^167^-induced NF-κB activation on HCC development, the NF-κB pathway inhibitor BAY-11–7082 was used in L02 cells expressing MHBSt^167^. The results showed that the intranuclear protein level of p-NF-κB/p65 was significantly increased in L02 cells expressing MHBSt^167^, while it paralleled level in L02 cells expressing wild-type MHBS in the presence of BAY-11-7082 (P < 0.05, Fig. 5A). Cell proliferation and the cell cycle were measured after L02 cells were transfected with MHBS and MHBSt^167^ expression plasmids and treated with BAY-11-7082. The results showed that the enhanced cell proliferation induced by MHBSt^167^ was abolished by BAY-11-7082 treatment (P < 0.05, Fig. 5B). The cell cycle was analyzed by FCM, and the results showed that the MHBSt^167^-induced increase in the proportion of G2/M-phase cells was abolished by BAY-11-7082 treatment (Fig. 5C, D, E), which suggests that MHBSt^167^-induced NF-κB activation accelerates cell cycle progression from S phase to G2/M phase.

### Autophagy promoted MHBSt^167^-induced NF-κB activation in L02 cells

**Fig. 6:**
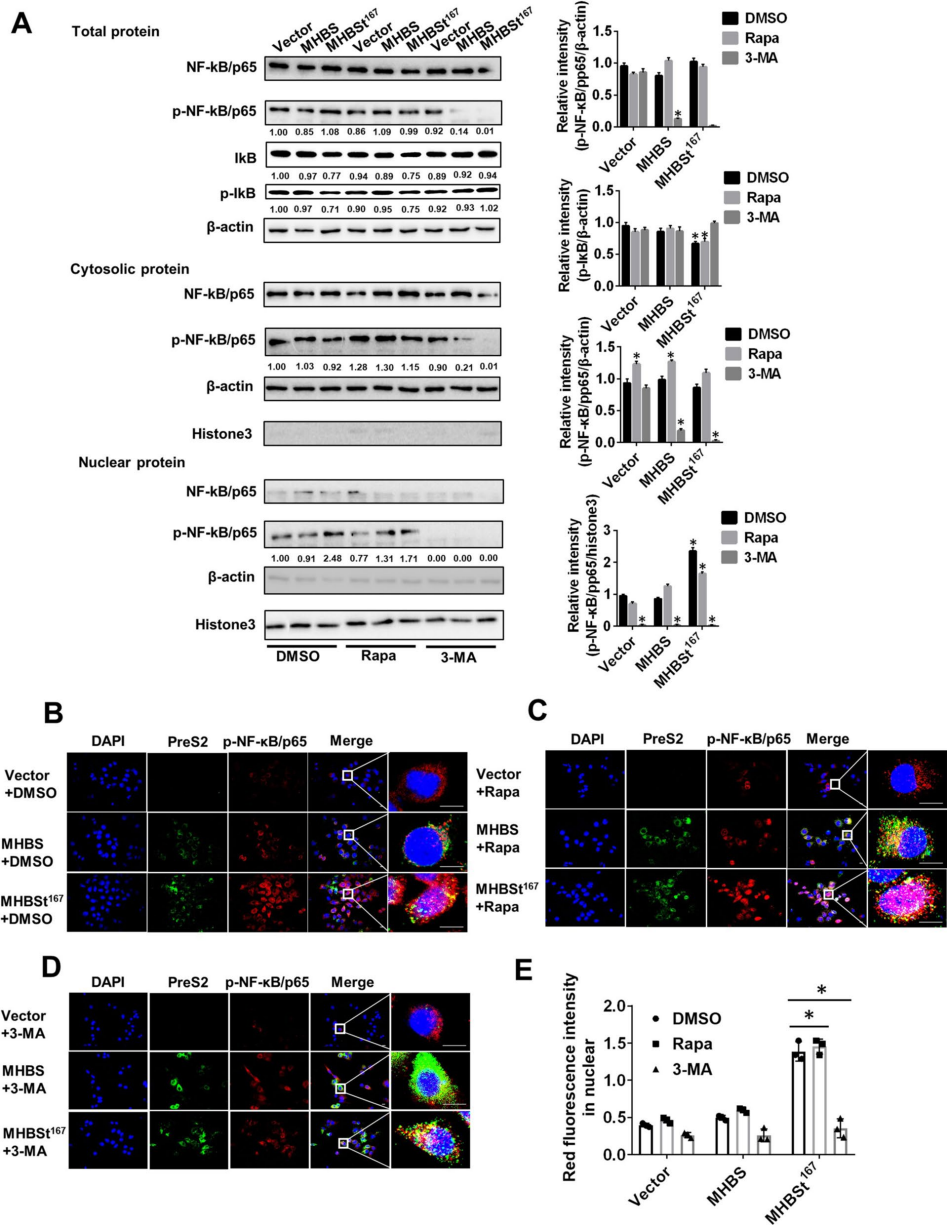
Effects of MHBSt^167^-induced autophagy on NF-κB activation in L02 cells. **A** Western blotting analysis of NF-κB activation and statistical analysis. Before transfection with MHBS and MHBSt^167^ expression plasmids, cells were treated with DMSO, 100 nM Rapa and 2 mM 3-MA for 2 h. The individual gray value in Western blots was measured and normalized against β-actin, then the relative intensity of target protein to β-actin was calculated by setting the control vector transfection as 1.00, *P < 0.05. **B**–**D** Confocal microscopic analysis of NF-κB activation. Before transfection with MHBS and MHBSt^167^ expression plasmids, cells were treated with DMSO, 100 nM Rapa and 2 mM 3-MA for 2 h. The intracellular localization of p-NF-κB/p65 was determined by immunofluorescence staining using an anti-p-NF-κB/p65 antibody. Nuclei were stained with DAPI. Scale bar: 50 μm. **E** Analysis of red fluorescence intensity in nuclear in B, C, D. Representative images from three independent experiments are shown

To explore the relationship between autophagy and the immune response induced by MHBSt^167^, the autophagy activator Rapa and the autophagy inhibitor 3-MA were used. The results showed that the protein level of p-NF-κB/p65 was hardly changed by Rapa treatment in MHBSt^167^ expressed cells, while it was significantly decreased by 3-MA treatment in the total protein, cytosolic protein and nuclear protein fractions (P < 0.05, Fig. 6A). The protein level of p-IκB was slightly increased by Rapa treatment and was significantly increased by 3-MA treatment. Immunofluorescence results showed that the nuclear level of p-NF-κB/p65 protein was scarcely changed by Rapa treatment, and it was significantly decreased by 3-MA treatment in cells expressing MHBSt^167^ (Fig. 6B–E), which indicated that the MHBSt^167^-induced activation of NF-κB was significantly inhibited by 3-MA treatment. To confirm the truncated MHBS protein induced NF-KB activation through autophagy, we used the ATG5-specific siRNA to inhibit autophagy specifically, and then investigate whether NF-κB was inhibited to verify our hypothesis. The result showed that NF-κB activation induced by MHBSt^167^ was abolished by siATG5 treatment (Additional file 2: Fig. S2A, B).

## Discussion

The present study showed that MHBSt^167^ could promote HCC development by promoting cell proliferation and EMT and accelerating cell cycle progression from S phase to G2/M phase. These findings are consistent with the results of an epidemiologic study and a report showing that MHBSt^167^ can enhance the proliferative activity of hepatocytes and upregulate oncogene expression. In addition, the present study showed that MHBSt^167^-induced autophagy and the NF-κB mediated innate immune response were related to HCC development.

It has reported that the preS mutants can activate both endoplasmic reticulum (ER) stress-dependent and ER stress-independent signals [5]. In no ER stress dependent manner, the preS mutants can additionally promote hepatocyte proliferation by inducing an ER stress-independent activation of a signal transduction pathway that involves the Jun activation domain-binding protein 1 (JAB1), the cyclin-dependent kinase (Cdk) inhibitor p27 [21]. After p27 inhibition, activation of CdK2 was upregulated and binding to Cyclins A to promote phase S to G2/M to inducing cell proliferation [22, 23]. In ER stress dependent manner, ER stress resulted in the activation of NF-κB and the calcium-dependent protease μ-calpain. The activation of μ-calpain in turn causes the cleavage of cyclin A resulting in an N-terminus-truncated product and promoting the binding of Cyclin A and CdK2 to accelerate cell cycle transition from S to G2/M [24]. PreS activate NF-κB signaling pathway to upregulate cyclooxygenase-2 (COX-2), vascular endothelial growth factor (VEGF) and human telomerase reverse transcriptase (hTERT) expression [5]. In addition, a truncated form of preS2 protein appears to be able to directly interact with a preS2-responsive DNA region and can activate the hTERT promoter, resulting in the upregulation of telomerase activity and in the promotion of HCC development. In this study, EMT and accelerated cell cycle transition from S to G2/M was induced by MHBSt^167^, we concluded that MHBSt^167^ contributes to HCC. In addition, MHBSt^167^ was retained within cells and the co-location of MHBSt^167^ and ER markers was significantly increased, which indicated that MHBSt^167^ induced HCC in ER stress dependent manner.

It has been reported that about 11% of secreted proteins and 20% of single-point or multipoint transmembrane proteins are inserted into the ER lumen by N-terminal signal sequences [25], 26. Misfolded or mutant proteins accumulate in ER and Ca^2+^ levels change could induce ER stress [27]. MHBS is inserted into the ER by three transmembrane structures, and the C-terminus is inserted into the ER membrane. However, the C-terminus of MHBSt^167^ is untethered in the ER, inducing ER stress [8]. ER stress triggers the unfolded protein response (UPR) to counteract the deleterious consequences of ER stress and restore ER homeostasis [28]. Autophagy is thought to be mainly related to UPR, promoting the survival of stress cells by removing unfolded proteins. The UPR and autophagy are two different programs related to cellular homeostasis, either working independently or working in cooperation to protect cell physiology from multiple stressors. In this study, MHBSt^167^ significantly increased autophagic flux. Autophagy in liver participates in functional biosynthesis and damaged organelles recycling [29]. Autophagy also plays an important role in Hepatic pathologic changes and tumor development. Abnormal autophagy results in oxidative stress, leading to abnormal gene expression, and could transform the cells, promoting tumorigenesis [30]. Autophagy acts as an anticancer mechanism, inhibiting the malignant transformation of normal cells into cancer cells [31]. On the other hand, autophagy is also implicated in different stages of cancer development and metastasis. The survival of fast-growing tumors is particularly correlated with their autophagic activity. The precise role of autophagy in HCC is unclear and has not been fully elucidated. The results of this study showed that the inhibition of autophagy could abolish MHBSt^167^-induced L02 cell proliferation and the cell cycle. This finding indicated that MHBSt^167^-induced autophagy could promote HCC development.

C-terminally truncated surface proteins are produced by the chromosomal integrated HBV sequences and nonintegrated viral variants, which formed by the selective pressure of the host immune response and/or antiviral treatments [16, 32]. This finding suggests that MHBSt^167^ may be related to the host immune response. To verify this hypothesis, NF-κB activation and the expression of downstream cytokines was examined. The results revealed significantly enhanced translocation of p-NF-κB/p65 from the cytoplasm to the nucleus in the presence of MHBSt^167^, and the expression of IFN-α, IFN-β and IL-1α was upregulated, indicating that MHBSt^167^ could induce an immune response. Cirrhosis and chronic inflammation are highly likely to develop into liver cancer, and its immunological mechanisms had been deciphered [33]. During the progression of liver diseases, immune and inflammatory responses are considered driving factors and prerequisites for liver cancer [34]. Factors that drive inflammation to develop into live cancer include abnormal regeneration after hepatocyte death, fibrosis, or angiogenesis [35]. However, malignant tumors also produce an intrinsic inflammatory response, which in some cases is beneficial for the antitumor response [36, 37]. BAY-11-7082, an NF-κB inhibitor, was used to verify whether the MHBSt^167^-induced immune response was related to HCC. The results showed that MHBSt^167^ enhanced cell proliferation and cell cycle, which could be blocked by BAY-11-7082 treatment. Taken together, our results indicate that MHBSt^167^ can promote HCC development by inducing the immune response.

It has been reported that a large number of immune-related signaling molecules can regulate autophagy, which suggests the central importance of autophagy in immunity. Autophagy is regulated by different immune-related signaling molecules, including pathogen-recognition receptors, pathogen receptors, downstream immunity-related GTPases, inhibitor of NF-κB (IKK) and NF-κB [38]. On the other hand, autophagy-related proteins can regulate innate immune signaling pathways. Type I IFN production was upregulated by autophagy-related proteins in dendritic cells while the RIG-I-like receptor-mediated induction of type I IFN production was negatively regulated by these proteins [39–41]. The autophagy protein ATG9A negatively regulates the activation of STING to inhibit the efficient activation of type I IFN and pro-inflammatory cytokine production in response to stimulatory DNA [42]. The innate immune signal molecule has its unique corresponding autophagy protein. The autophagy pathway and/or proteins also play decisive roles in regulating inflammatory responses. In autophagy-deficient cells, p62 expression was increased and accumulated in cells, leading to the activation of the pro-inflammatory transcription factor NF-κB through a mechanism involving TRAF6 oligomerization [43]. In hepatocytes deficient in the autophagy protein Atg7, the accumulation of p62 leads to enhanced activation of the stress-responsive transcription factor NRF2 and liver injury [38]. Melanoma patients and mouse models have upregulated autophagy level, tumor growth was decreased in myeloid cells and antitumor immune response was induced in vivo by inhibiting autophagy. In myeloid-derived suppressor cells, expression of membrane-associated RING-CH1 E3 ubiquitin ligase was decreased by inhibiting autophagy, leading to enhanced surface expression of MHC-II and followed by tumor-specific CD4 T cells expansion [44]. Expression level and release of the cytokine CCL5 were increased by inhibition of BECN1 via the MAPK8/JNK-JUN/c-Jun signaling pathway, resulting in tumor growth inhibition and massive natural killer cell infiltration into the tumor microenvironment (45). Overall, these findings emphasize the importance of autophagy in the tumor immune response. To explore the relationship between autophagy and the immune response induced by MHBSt^167^, 3-MA and ATG5 siRNA was used to inhibit autophagy. In the present study, MHBSt^167^ induced an immune response, as evidenced by the enhanced translocation of p-NF-κB/p65 from the cytoplasm to the nucleus, and MHBSt^167^-induced p-NF-κB/p65 nuclear translocation was significantly inhibited after autophagy was inhibited with 3-MA and siATG5. It is incongruent with the findings that autophagy activation by rapamycin itself does not induce any NF-κB activation nor oncogenic phenotype in L02 cells. We speculate that autophagy activation was not the only pathway related to MHBSt^167^-mediated NF-κB activation and subsequent oncogenic phenotype. The other pathway activated by MHBSt^167^ and autophagy may work together to be responsible for MHBSt^167^-mediated NF-κB activation and subsequent oncogenic phenotype.

This study revealed a novel mechanism of HBV-related HCC. C-terminal truncation of MHBS that occurs during persistent HBV infection can induce an immune response and autophagy and contribute to the development and progression of HCC. Since the present study used an immortalized cell line, further study in non-immortalized cells and in vivo experiments might be needed to verify the carcinogenic mechanism of MHBSt^167^. Furthermore, as the mutation in the S region that leads to C-terminally truncated MHBS may generate C-terminally truncated LHBS and SHBs as well, the latter two truncated surface proteins may act synergistically with MHBSt in the development of HCC.

## Conclusion

Taken together, the present study demonstrated that MHBSt^167^ could promote the development of HCC by inducing cell proliferation, accelerating cell cycle progression from S phase to G2/M phase, and promoting EMT. Autophagy could enhance the MHBSt^167^-induced host immune response, and the interplay of autophagy with the MHBSt^167^-induced immune response may contribute to the development and progression of HCC.

## Supplementary Information

**Additional file 1**.
Figure S1. Effects of MHBSt^167^ expression on cell proliferation, cell cycle and EMT in L02 and hepatoma cell lines. A) The RT-PCR products analysis. Lane 1, lane 2, mRNA from cells transfected with pcDNA3.1‑MHBSt^167^and pcDNA3.1‑MHBS, respectively. M, DL2000 DNA marker. B) Western blotting analysis of protein expression. Cells were transfected with different amounts of pcDNA3.1‑MHBS (0.6, 0.8, 1.2 and 2.4 µg) and 2.4 µg of pcDNA3.1-MHBSt^167^. C) Cell viability was measured by CCK-8 assays after being transfected with plasmids for 48 hours. The results are expressed as OD values, *P < 0.05. D)Cell cycle was analyzed by flow cytometry. Cells were stained with PI after being transfection for 48 hours. E) Western blotting analysis of Vimentin and E-cadherin protein expression. The individual gray value in Western blots was measured and normalized against β-actin, then the relative intensity of target protein to β-actin was calculated by setting the control vector transfection as 1.00, *P < 0.05.F) Ponceau stain of the membrane of western blotting analysis of MHBSt^167^ in culture supernatants and cellular extracts. Representative images from three independent experiments are shown.

**Additional file 2**.
Figure S2. Effects of siATG5 on NF-κB activation induced by MHBSt^167^ in L02 cells. A) Western blotting analysis of NF-κB activation and statistical analysis in total protein. B) Western blotting analysis of NF-κB activation in nuclear protein and cytosolic protein and statistical analysis. SiATG5 was co-transfected with MHBS and MHBSt^167^ expression plasmids for 24h. The individual gray value in Western blots was measured and normalized against β-actin, then the relative intensity of target protein to β-actin was calculated by setting the control vector transfection as 1.00, *P < 0.05.

## Data Availability

Not applicable.

## Abbreviations

HCC: Hepatocellular carcinoma
IFN-α: Interferonα
IFN-β: Interferonβ
IL-1α: Interleukin-1α
ER: Endoplasmic reticulum
NF-κB: Nuclear factor-κB
ROS: Reactive oxygen species
PDTC: Pyrimidine dithiocarbamate
HCS: High-content screening
FCM: Flow cytometry
EMT: Epithelial-mesenchymal transition
Rapa: Rapamycin
CQ: Chloroquine
3-MA: 3-Methyl adenine
JAB1: Domain-binding protein 1
Cdk: The cyclin-dependent kinase
COX-2: Cyclooxygenase-2
VEGF: Vascular endothelial growth factor
hTERT: Human telomerase reverse transcriptase
MHC-II: Histocompatibility complex-II
