# Supplementary figures and images for: MHBSt167 induced autophagy promote cell proliferation and EMT by activating the immune response in L02 cells
Source: Virol J. 2022 Jun 27;19:110. doi: 10.1186/s12985-022-01840-z (PMC9235077; doi:10.1186/s12985-022-01840-z)

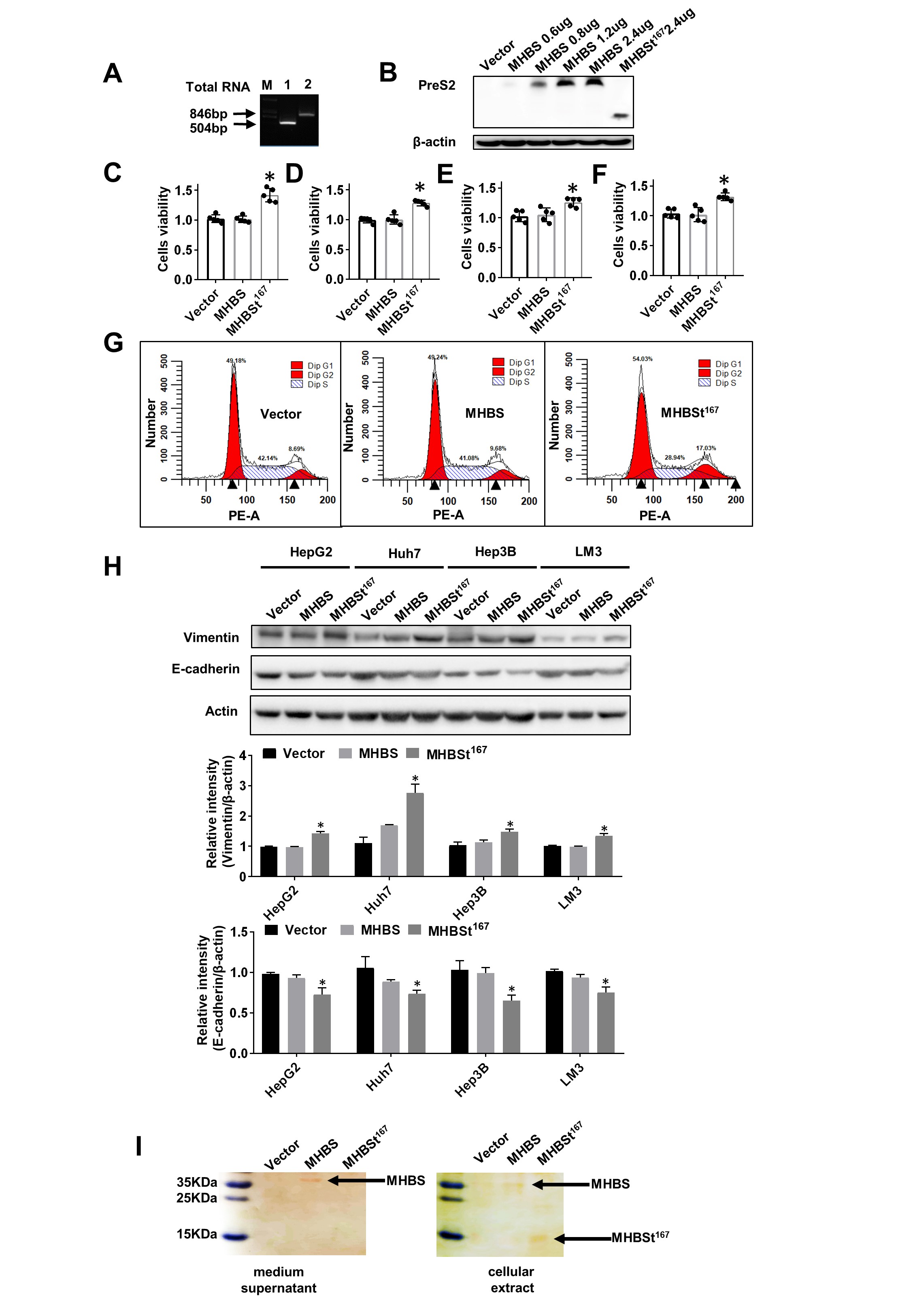

Supplement: Supplementary file 1 — Additional file 1. Figure S1. Effects of MHBSt167 expression on cell proliferation, cell cycle and EMT in L02 and hepatoma cell lines. A) The RT-PCR products analysis. Lane 1, lane 2, mRNA from cells transfected with pcDNA3.1‑MHBSt167and pcDNA3.1‑MHBS, respectively. M, DL2000 DNA marker. B) Western blotting analysis of protein expression. Cells were transfected with different amounts of pcDNA3.1‑MHBS (0.6, 0.8, 1.2 and 2.4 µg) and 2.4 µg of pcDNA3.1-MHBSt167. C) Cell viability was measured by CCK-8 assays after being transfected with plasmids for 48 hours. The results are expressed as OD values, *P < 0.05. D)Cell cycle was analyzed by flow cytometry. Cells were stained with PI after being transfection for 48 hours. E) Western blotting analysis of Vimentin and E-cadherin protein expression. The individual gray value in Western blots was measured and normalized against β-actin, then the relative intensity of target protein to β-actin was calculated by setting the control vector transfection as 1.00, *P < 0.05.F) Ponceau stain of the membrane of western blotting analysis of MHBSt167 in culture supernatants and cellular extracts. Representative images from three independent experiments are shown. [file 12985_2022_1840_MOESM1_ESM.jpg]

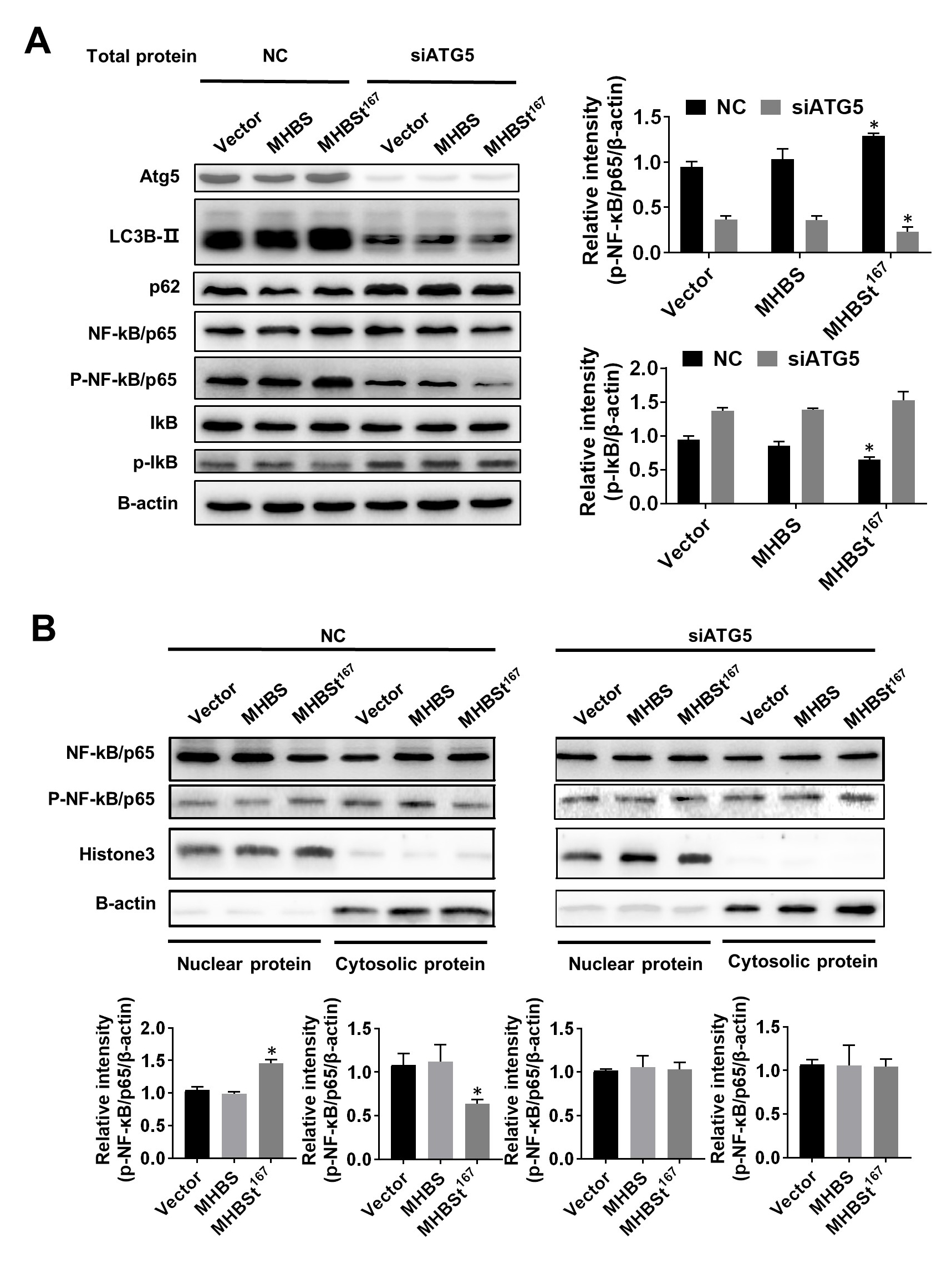

Supplement: Supplementary file 2 — Additional file 2. Figure S2. Effects of siATG5 on NF-κB activation induced by MHBSt167 in L02 cells. A) Western blotting analysis of NF-κB activation and statistical analysis in total protein. B) Western blotting analysis of NF-κB activation in nuclear protein and cytosolic protein and statistical analysis. SiATG5 was co-transfected with MHBS and MHBSt167 expression plasmids for 24h. The individual gray value in Western blots was measured and normalized against β-actin, then the relative intensity of target protein to β-actin was calculated by setting the control vector transfection as 1.00, *P < 0.05. [file 12985_2022_1840_MOESM2_ESM.jpg]
